# Ascites-derived CDCP1+ extracellular vesicles subcluster as a novel biomarker and therapeutic target for ovarian cancer

**DOI:** 10.3389/fonc.2023.1142755

**Published:** 2023-07-03

**Authors:** Lingnan Kong, Famei Xu, Yukuan Yao, Zhihui Gao, Peng Tian, Shichao Zhuang, Di Wu, Tangyue Li, Yanling Cai, Jing Li

**Affiliations:** ^1^ Affiliated Hospital of Weifang Medical University, School of Clinical Medicine, Weifang Medical University, Weifang, China; ^2^ Department of Pathology, Zibo Central Hospital, Zibo, China; ^3^ Department of Ultrasonic, Zibo Central Hospital, Zibo, China; ^4^ Department of Gynecology, Zibo Central Hospital, Zibo, China; ^5^ Department of R&D, Shenzhen SecreTech Co., Ltd., Shenzhen, China; ^6^ Department of R&D, Vesicode AB, Solna, Sweden; ^7^ Guangdong Provincial Key Laboratory of Systems Biology and Synthetic Biology for Urogenital Tumors, The First Affiliated Hospital of Shenzhen University, Shenzhen Second People’s Hospital, Shenzhen, China

**Keywords:** extracellular vesicles, CDCP1, ovarian cancer, ascites, diagnose

## Abstract

**Introduction:**

Ovarian cancer (OVCA) is one of the most prevalent malignant tumors of the female reproductive system, and its diagnosis is typically accompanied by the production of ascites. Although liquid biopsy has been widely implemented recently, the diagnosis or prognosis of OVCA based on liquid biopsy remains the primary emphasis.

**Methods:**

In this study, using proximity barcoding assay, a technique for analyzing the surface proteins on single extracellular vesicles (EVs). For validation, serum and ascites samples from patients with epithelial ovarian cancer (EOC) were collected, and their levels of CDCP1 was determined by enzyme-linked immunosorbent assay. Tissue chips were prepared to analyze the relationship between different expression levels of CDCP1 and the prognosis of ovarian cancer patients.

**Results:**

We discovered that the CUB domain-containing protein 1+ (CDCP1+) EVs subcluster was higher in the ascites of OVCA patients compared to benign ascites. At the same time, the level of CDCP1 was considerably elevated in the ascites of OVCA patients. The overall survival and disease-free survival of the group with high CDCP1 expression in EOC were significantly lower than those of the group with low expression. In addition, the receiver operating characteristic curve demonstrates that EVs-derived CDCP1 was a biomarker of early response in OVCA ascites.

**Discussion:**

Our findings identified a CDCP1+ EVs subcluster in the ascites of OVCA patients as a possible biomarker for EOC prevention.

## 1 Introduction

Ovarian cancer (OVCA) is a prevalent kind of malignant tumor in the female reproductive system, and it has the highest mortality rate of all gynecological malignancies (1, 2). Epithelial ovarian cancer (EOC) accounts for approximately two-thirds of OVCA and is the most prevalent and fastest-progressing pathological subgroup (3, 4). OVCA is characterized by the development of ascites within the peritoneal cavity. Tumor cells of OVCA in ascites have the characteristics of epithelial-mesenchymal transitions that can be transformed into more invasive spindle cells. Therefore, it is completely reasonable to think that the production of malignant ascites should be closely relevant to high invasiveness of OVCA. It correlates with metastases and, hence, a dismal prognosis for OVCA (5).

Liquid biopsy (6), used for early screening, diagnosis, and prognosis, is an effective method for guiding treatment and reducing tumor mortality. Currently, serum tumor markers such as carbohydrate antigen 125 (CA125), carcinoembryonic antigen, α-Fetoprotein and human chorionic gonadotropin in conjunction with color doppler ultrasonography and computed tomography (7, 8), are frequently employed for OVCA diagnosis (9). However, the sensitivity and specificity of these tumor markers are inadequate for clinical applications, necessitating the search for new diagnostic or therapeutic targets.

Exosomes are extracellular vesicles (EVs) secreted by the majority of cells. It has been found that exosomes contain a variety of bioactive proteins, such as major histocompatibility complex I (MHC I) and major histocompatibility complex II (MHC II) involved in antigen presentation, as well as transmembrane proteins and annexins (10). Exosomes transport proteins, DNA and non-coding RNA from the host cells (11, 12). Cancer-derived exosomes have been demonstrated to facilitate cancer progression by stimulating angiogenesis, pre-conditioning of metastatic locations, suppressing immune systems and others (13). Exosomes have been demonstrated in the ascites of both OVCA patients (14) and non-cancerous patients with liver or kidney disorders. According to previous research, exosomes produced from ascites in OVCA patients have been demonstrated to correlate strongly with tumor burden, invasiveness, and poor prognosis. Meanwhile, ascites-derived exosomes can contribute to cancer growth by altering the tumor microenvironment, which alters cancer cells’ biological characteristics and functions and various tumor cell behaviors (11). Therefore, the search for disease-specific exosome-associated markers has the potential to reveal the mechanism of peritoneum metastasis of OVCA and to propose diagnostic or therapeutic targets for OVCA (15). However, an important concern remains that diagnostic surface protein markers of exosomes may be too rare in abundance to be detected in the sample. Therefore, how to accurately analyze the composition of complex exosomes in ascites and obtain more effective information from ascites related exosomes for accurate diagnosis and treatment of diseases has become an urgent technical problem to be solved.

In this investigation, proximity barcoding assay (PBA) (16), a single-EVs analysis method developed by our collaborating group, was utilized to profile the expression of CDCP1 proteins at single EVs and followed by bioinformatic analysis for the identification of exosome subpopulation. Based on the results of PBA, the positive marker in ascites-derived EVs, CUB domain-containing protein 1 (CDCP1), was selected for further investigation.

CDCP1 (17) is a transmembrane protein that has been previously proven to be closely associated with the occurrence of colorectal cancer (18), lung cancer (19), breast cancer (20), prostate cancer (21) and other diseases (22, 23). The above studies have shown that CDCP1often is regard as an important hub for oncogenic signaling. It suggests that CDCP1 could be a promising and widely used biomarker for early diagnosis and prognosis. It was discovered that the proportion of CDCP1+ EVs in OVCA ascites was significantly elevated, indicating that EVs-derived CDCP1 can be considered a molecular biomarker for early surveillance and diagnosis of OVCA.

## 2 Materials and methods

### 2.1 Ethical approval, patient recruitment and sample collection

The study was approved by the Medical Ethics Committee of Zibo Center Hospital. Between Jan 2019 and Dec 2021, 8 patients with high-grade serous OVCA and 18 patients with non-cancer-disease-induced ascites were enrolled in our study at Zibo center Hospital, including patients with chronic kidney disease and heart failure. Non-cancer women were selected in the control group. Ensure that there is no history of ovarian cancer or other tumors, o abdominal or pelvic space occupation in imaging, and the expression of serological tumor markers is in the normal range. Only when these conditions are met at the same time can they be included in the control group. The ovarian cancer group selected patients who were diagnosed with EOC with ascites and did not receive radiotherapy or chemotherapy before surgery. After ascites samples were collected, they were centrifuged at 1,000 × g at 4°C for 15 min (R5810, Eppendorf, Germany) to remove cells and then at 10,000 × g at 4°C for 30 min (R5810, Eppendorf, Germany) to remove cell debris or aggregates.

Ultracentrifugation (Optima XPN100, Coulter Beckman with rotor SW45Ti) steps were performed at 100, 000 × g at 4°C for 70 min to precipitate EVs, followed by a washing step with phosphate buffered saline (PBS) and a second ultracentrifugation step at 100,000 × g at 4°C for 70 min. The pellets from 10 ml of ascites were resuspended in 100 µL PBS for further analysis. The size distribution and concentration of exosome suspension were analyzed via nanoparticle tracking analysis (NTA, nanosight NS300, Malvern Panalytical, UK). The EVs captured on analyzing surface were recorded via Scanning Electron microscopy (SEM, Zeiss, Germany).

### 2.2 Proximity barcoding assay and sequencing

To characterize the single EVs isolated from the ascites of OVCA and non-cancerous patients. We used proximity barcoding assay (PBA) to detect the expression of 113 proteins (**Supplementary Table 1**) on individual EVs. The majority of proteins examined are known cancer biomarkers. The analysis was performed following the standard operating procedures defined by Vesicode (Solna, Sweden) and reported in our earlier paper (16). Briefly, antibodies labeled with designed DNA probes were utilized to identify exosomal proteins concurrently. The DNA probes comprise protein tags, molecular tags and universal binding sites to subsequent processes. With the assistance of EV barcoding templates, EV tags were attached to the 3’ end of DNA probes to label the antibodies recognizing the same single EVs. Therefore, we generate DNA sequences comprising EV tag, protein tag and molecular tag. After library preparation, the sequencing was conducted using the Illumina NextSeq 500 platform with 75 cycles of single-end sequencing.

### 2.3 Exosomal proteome data analysis

The sequencing data were generated with high-throughput sequencing. The bcl2fastq software (version 2.20.0.42, Illumina Inc., USA) was used to convert raw data into fastq files of each sample. The fastQC technique was utilized to assess the read quality (24). The fastp technique controls read quality and adapter trimming (25). Reads with a quality score < 20 were eliminated. Sequencing reads comprising the EV tag, protein tag and molecular tag were evaluated to build a matrix of EV ID and protein expression data for each sample, which provides a high-dimensional protein abundance dataset at the single EV level for subsequent analysis.

The sum of protein counts from all single EVs in a sample was regarded as the raw protein expression data for assessing each protein’s expression level in each sample. The raw protein expression values were normalized utilizing the trimmed mean of M-values (TMM) algorithm in the edgeR package (26).

The number of EVs with each pair of co-expressed proteins was counted as a protein combination dataset for analyzing the pattern of protein-protein combinations on individual EVs. The count per million (CPM) normalization procedure was applied prior to further examination of combination data. To examine the performance of EV proteins as a biomarker in the classification of patients and controls, we construct the receiver operating characteristic (ROC) curves and generate the area under the receiver operating characteristic curve (AUC) (27)values using the pROC package (v. 1.18.0). For EV subpopulation analysis, the unsupervised FlowSOM algorithm (28)was utilized to generate EV clusters. T-distributed stochastic neighbor embedding (t-SNE) was used for subpopulation visualization (29). The analyzes were conducted in R version 4.0.5.

### 2.4 Tissue chip, hematoxylin and eosin staining and immunohistochemical staining

Tissue chips of OVCA and control tissues were obtained from Shanghai Outdo Biotech Company (Shanghai, China; Cat # HOvaC154Su01). Before surgery, no treatment was given to patients pathologically diagnosed with OVCA. The antibody employed for IHC was Anti-WT-1 (dilution 1:100; cat. no. IR346; LBP, Guangzhou, China). Anti-CA125 (dilution 1:100; cat. # IM360; LBP, Guangzhou, China). Anti-ki67 (dilution 1:100; cat. # 790-4286; Roche, Basel, Switzerland). Tissues were cut into 2-3 µm slices for future usage, baked at 60°C for 30-60 minutes, and repaired at 92-95°C for half an hour. The first antibody and the second antibody were added after washing with PBS. The color was developed by diaminobenzidine at 37°C for 40 min. CDCP1 IHC was performed using an antibody against CDCP1 (Rabbit monoclonal to CDCP1-C-terminal; dilution 1:100; Abcam, ab252947; USA), as previously described.

Immunostaining was reviewed by two pathologists. However, clinical information was not visible to them. Scoring was performed by two experienced pathologists who were blinded to tissue identity using a grading system based on staining intensity (no staining, 0; weak, 1; moderate, 2; strong, 3) and percentage of positive-staining cells (1–25% positive, 1; 26–50%, 2; 51–75%, 3; 76–100%, 4) (30, 31). The final score was calculated as intensity score × percentage score. For survival analysis, the final score categorized CDCP1 expression in OVCA tissues as low (0–4) or high (5–12). Based on CDCP1 expression in tissue chips of 79 patients, Kaplan–Meier survival curves were constructed for high- and low-expression groups and assessed with a log-rank test (*p* < 0.05).

### 2.5 Bioinformatics analysis

The Cancer Genome Atlas (TCGA, https://cancergenome.nih.gov/) is a public project aimed at cataloging and discovering major carcinogenic genomic changes in the progression of human tumors by large-scale genome sequencing and thorough multidimensional analysis (32). The Gene Expression Profiling Interactive Analysis (http://gepia.cancer-pku.cn/index.html) is a recently developed bioinformatics platform that contains tissue expression data from 9736 tumors and 8587 normal samples. Making cancer genome data sets accessible to the public makes it possible to improve diagnostic methods, treatment criteria, and cancer prevention. In this study, “expression DIY” was used to compare CDCP1 in OVCA with control tissues in TCGA database, with *p* < 0.05 as the significant criterion for screening.

### 2.6 Enzyme-linked immunosorbent assay

Serum samples and ascites of patients with EOC were preserved at -80°C until use. Each sample was centrifuged for 20 min at 2,000 rpm and 4°C, and then the supernatant liquid was collected. CDCP1 was assessed with human-specific sandwich ELISA kits per the manufacturer’s instruction (FMGBio, shanghai, China).

The final ELISA assay was performed as follows: washing buffer consists of 1.5 mM NaH2PO4, 8.5 mM Na2HPO4, 145 mM NaCl, 1 g/L Tween 20, deionized H2O, and pH 7.4. Then, 50 µL assay buffer was added to each well. First, 50 µL of either sample or control was added to each well. The calibrators are prepared by adding 50 µL assay buffer beforehand. Each well was then filled with 50 µL of biotinylated antigen solution. After incubation for 0.5 h at 37°C, the plates were washed five times with washing buffer before 50 µL of horseradish peroxidase-avidin was added to each well. Incubate at 37°C for another 0.5 h. The plates were rinsed five times. Add 50 µL of chromogenic agent A and 50 µL of chromogenic agent B to each well, shake gently and mix well. Develop the color for ten minutes at 37°C, and add 50 µL of termination solution to each well to terminate the reaction. Each well’s optical density (OD) was measured at 450 nm on the MultiSkan Ascent (Thermo Scientific, Odense, Denmark) by setting the blank wells to zero. The measurement must be conducted within 10 min of adding the termination solution. The regression equation of the standard curve is produced from the concentration and OD value, and then the results of samples and controls are read from this curve.

### 2.7 Statistical analysis

We applied the Shapiro-Wilk test to examine the normality of data distribution and the F-test to determine the variance homogeneity. The student’s t-test was applied for differential expression analysis of groups with normal data distribution and homogenous variance. A non-parametric test was employed to compare protein expression between groups with unequal variance or abnormal distributions. The Mann-Whitney U test is applied if the data are not normally distributed. Welch’s t-test is used for data having normal distribution but nonhomogeneous variance. We employed the Benjamini-Hochberg (BH) method to adjust the *p*-values. Other statistical analyzes were processed using SPSS 23.0 software (IBM Corporation, Armonk, NY, USA) and GraphPad Prism 5.0 (GraphPad Software, La Jolla, CA, USA). When n ≤ 5 or the proportion of n ≤ 5 is more than 20%, the Spelman rank correlation coefficient is used. *p* < 0.05 indicated that a statistically significant difference exists.

## 3 Results

### 3.1 Characterization of EVs derived from ascites samples

**Figure 1:**
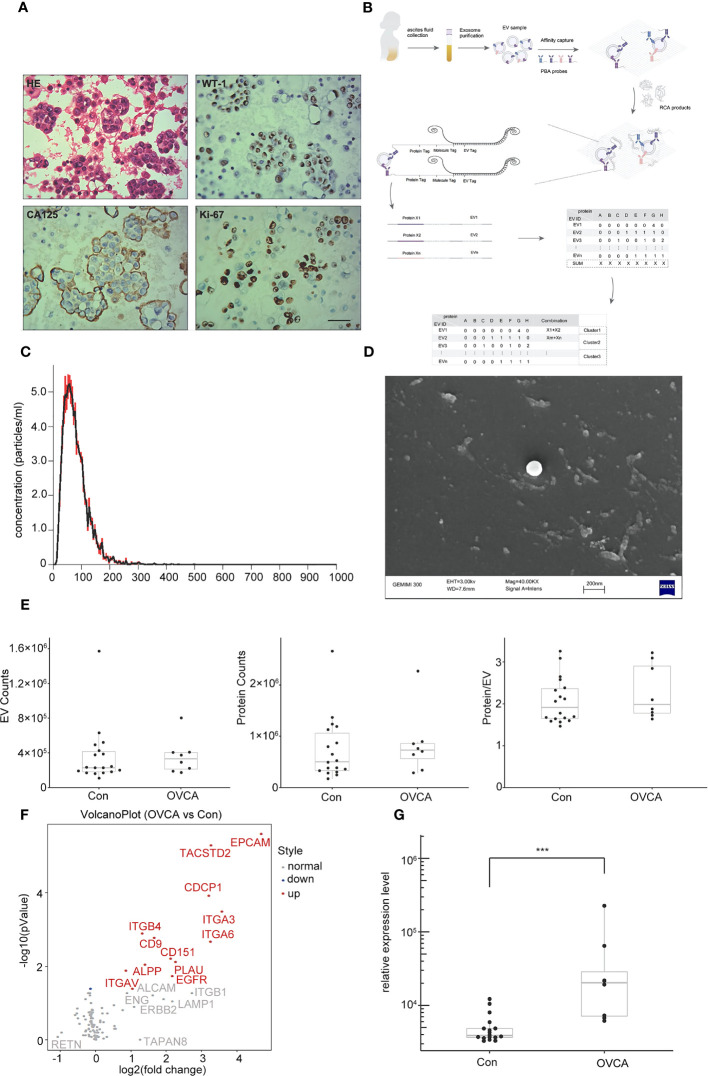
Extracellular vesicles (EVs) derived from ovarian cancer ascites samples. **(A)** Ovarian cancer (OVCA) tissue express WT-1, CA125 and ki67, scale bar 50 µm. **(B)** The protocol of Proximity Barcoding Assay (PBA) technology. **(C)** Exosomes were analyzed via nanoparticle tracking analysis. **(D)** Scanning Electron microscopy shows exosomes. **(E)** The count of EV and proteins. **(F)** Compared with the Con group, there were 12 upregulated, including CDCP1. **(G)** CDCP1 was shown higher expression in the OVCA group. ***p < 0.001.

By creating cellular wax blocks of ascites from OVCA patients, it was discovered that tumor cells distinctively express CA125 and WT-1 (**Figure 1A**). Next, we performed NTA and SEM characterization prior to PBA profiling of single EV level proteins (**Figure 1B**). The obtained EVs from ascites samples of patients were analyzed using NTA. The median size is 100 nm, and there is a concentration of 5 particles per mL (**Figure 1C**). SEM images of the collected EVs reveal a diameter of approximately 100 nm (**Figure 1D**). During PBA analysis, EVs were immobilized on a surface by the interaction between CTB and GM1 groups in the lipid membrane of EVs. **Figure 1E** depicts the number of observed protein counts, EV counts, and resultant protein counts per exosome. There was no significant difference between OVCA and control groups. Following PBA analysis, we evaluated the total expression level of each protein. We discovered that among 13 significantly differentially expressed proteins, 12 were upregulated in the OVCA group, and 1 protein was down-regulated (**Figure 1F**). Among the Upregulated proteins includes CDCP1, epithelial cell adhesion molecule (EPCAM), integrinα6 (ITGA6), human tumor-associated calcium signal transducer 2 (TACSTD2), integrinα3 (ITGA3), integrinβ4 (ITGB4), CD151, CD9 and like other. CDCP1 levels were statistically significantly higher in the OVCA group compared to the control group (**Figure 1G**).

### 3.2 The CDCP1 + EVs cluster was enriched in the ascites of OVCA patients

**Figure 2:**
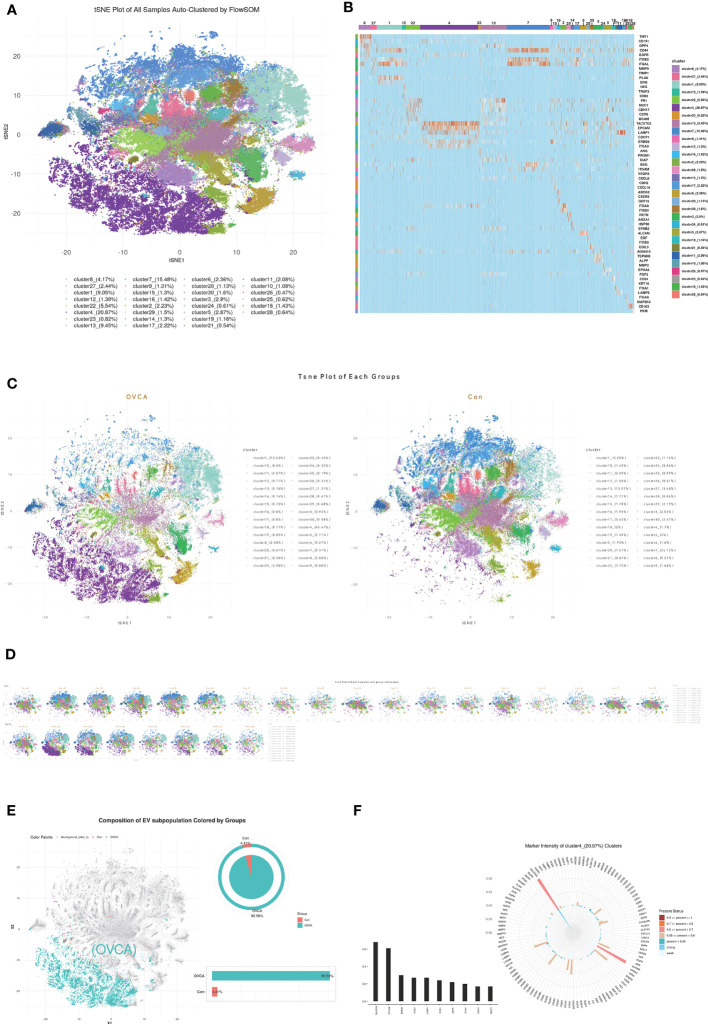
The CDCP1 + EVs cluster was enriched in the ascites of OVCA patients. **(A)** All samples were divided into twenty-eight clusters and demonstrated in the tSNE plot. **(B)** The Percentile of each subgroup and its characteristic protein. **(C)** EVs in ovarian cancer ascites and benign ascites. **(D)** tSNE of each sample with ovarian cancer and the control group. **(E)** OVCA ascites are mainly concentrated in cluster 4. **(F)** Major protein constituent of cluster 4.

We utilized FlowSOM, an unsupervised machine learning algorithm, to classify single EVs into clusters based on the similarity of their proteomic features. Twenty-eight clusters were obtained and are shown in the tSNE plot in **Figure 2A**. The Percentile of each subcluster and its representative protein are displayed in **Figure 2B**. To compare OVCA and control groups, we plotted the tSNE for each group (**Figure 2C**) and each sample (**Figure 2D**) and selected the subclusters with a significant difference. The plot of cluster 4 in **Figure 2E** depicts that the majority of the cluster of the EVs come from the OVCA group, i.e., 95.59%. The detection frequency of each protein in cluster 4 was plotted, and leading biomarkers include TACSTD2, EPCAM, erb-b2 receptor tyrosine kinase 2(ERBB2), ITGA3, lysosome associated membrane protein 1(LAMP1), integrinβ1(ITGB1), epidermal growth factor receptor (EGFR), ITGA6, CD151, CDCP1 (**Figure 2F**).

### 3.3 The correlation between CDCP1 expression level and clinical prognosis of EOC patients

**Figure 3:**
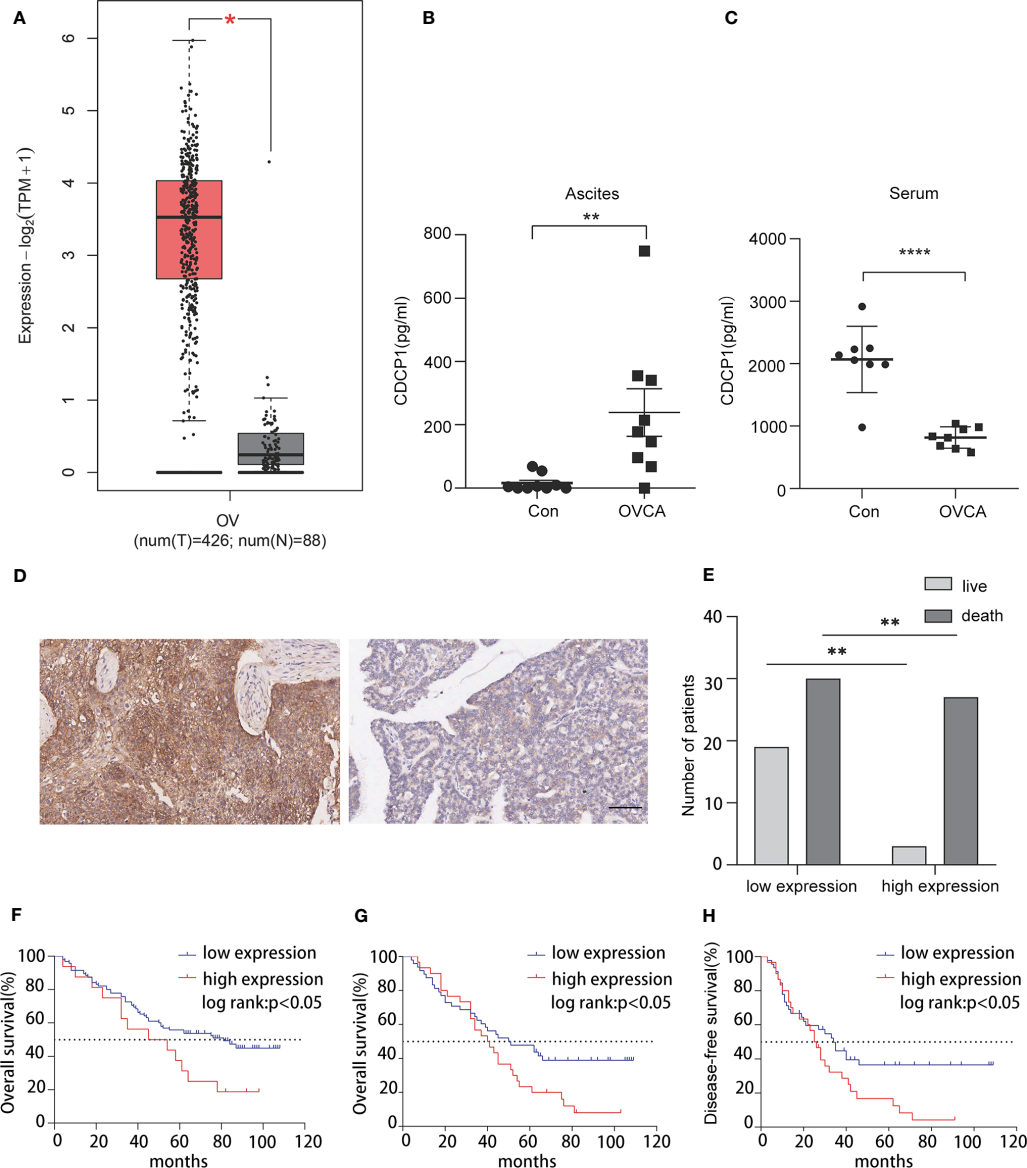
CDCP1 expression is associated with the prognosis of ovarian cancer patients. **(A)** CDCP1 expression level was positively linked with ovarian cancer in TCGA. **(B)** CDCP1 levels in the ascites were assessed using sandwich enzyme-linked immunosorbent assay (p < 0.05). **(C)** CDCP1 levels in the serum were assessed by the sandwich ELISA (p < 0.0001). **(D)** CDCP1 low and high expression as determined by IHC staining, scale bar 50 µm. **(E)** CDCP1 express level influenced survival. **(F)** Overall, greater ki-67 expression was associated with a shorter survival duration than low expression. **(G, H)** Overall and disease-free survival times of the group with high CDCP1 expression were significantly shorter than those with low CDCP1 expression. *p < 0.05, **p < 0.01, ****p < 0.0001.

TCGA database analysis revealed that the CDCP1 expression level was positively correlated with OVCA (**Figure 3A**). According to previous research, CDCP1 is highly expressed in OVCA tissues. In addition, we collected ascites and serum samples from women with EOC and non-cancer and determined the expression level of CDCP1 by enzyme-linked immunosorbent assay (ELISA). The findings revealed that the expression of CDCP1 was elevated in the exosomes of OVCA patients with ascites [n = 9, *p* < 0.05, 95% confidence interval (CI) 65.74 to 385.1] while decreasing in their serum (n = 8, *p* < 0.0001, 95% CI -1675 to -829.0) (**Figures 3B, C**). A microarray of OVCA tissue was created, and clinical survival data for OVCA patients were evaluated to investigate the influence of varying levels of CDCP1+ expression on disease-free survival or overall survival in patients with OVCA. According to the intensity and proportion of CDCP1 positive expression in the cytoplasm of tumor cells, CDCP1 was categorized into high and low expression groups (**Figure 3D**). The results showed that the survival rate of patients with low expression of CDCP1 was significantly higher than that of patients with high expression of CDCP1 (**Figure 3E**). The expression level of ki67 was also favorably linked with overall survival (n = 111, *p* < 0.05) (**Figure 3F**). From the clinical follow-up data, it is showed that the longest disease-free survival time and overall survival time of patients with ovarian cancer can reach 109 months. The results demonstrated that both the overall survival time (n = 78, *p* < 0.05) and the disease-free survival time (n = 76, *p* < 0.05) were significantly shorter in the high CDCP1 expression group than in the group with the low CDCP1 expression (**Figures 3G, H**). A comprehensive analysis of the expression level of CDCP1, clinicopathological grade and tumor TNM stage concluded that the expression level of CDCP1 varied significantly between different TNM stages (**Table 1**).

**Table 1:** The CDCP1 expression level varied between different stages.

Characteristic	High	Low	Total	rs	p-value
Age(years)					
≤50	9	21	30	0.117	0.306
>50	20	28	48		
Null			1		
Grades					
I	3	10	13	0.179	0.125
II	0	1	1		
III	27	34	61		
Null			4		
T Stage					
T1	0	1	1	0.228	0.043*
T2	2	11	13		
T3	28	37	65		
N Stage					
N0	15	31	46	0.131	0.252
N1	15	18	33		
M Stage					
M0	20	32	52	-0.014	0.903
M1	10	17	27		
TNM stage					
I	0	1	1		
II	2	11	13	0.098	0.389
III	18	20	38		
IV	10	17	27		

*p < 0.05.

### 3.4 The diagnostic value of single EV protein biomarkers in OVCA

**Figure 4:**
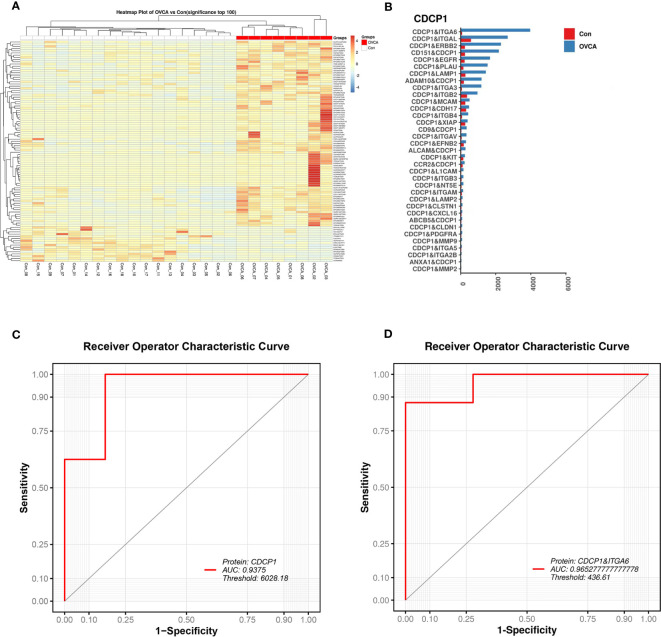
The diagnostic value of single EV protein biomarkers. **(A)** Multiple protein combinations are created in the OVCA group. **(B)** Characteristic protein combination constituted of CDCP1 in the OVCA group. **(C)** The receiver operating characteristic (ROC) curve for CDCP1. **(D)** The ROC curve for the combination of proteins CDCP1 and ITGA6.

The protein combination pattern between the OVCA and Control groups differs significantly. OVCA group displays mostly highlighted protein combinations among the combinations we discovered (**Figure 4A**). CDCP1 protein tends to co-express with ITGA6, ITGAL, ERBB2, and other proteins (**Figure 4B**). CDCP1 levels are significantly elevated. Using CDCP1 as a diagnostic biomarker, the AUC value in the ROC analysis is 0.9375 (**Figure 4C**). The combination of CDCP1 and ITGA6 proteins may differentiate between OVCA and non-cancer groups with a higher AUC value (0.9653) than CDCP1 alone (**Figure 4D**).

## 4 Discussion

CA125 is the main tumor marker for the diagnosis of EOC, but which is negative in some cases of EOC and affected by menstruation, pelvic inflammation, gynecological benign tumors and so on. As a new biomarker, human epididymis protein 4 has been used in the diagnosis of OVCA, but its expression varies greatly in different subtypes, so its clinical application is limited. The combined detection of multiple markers may improve the clinical application value of these markers (33), but more biomarkers does not mean more benefits (34). It is necessary to make a comprehensive judgment combined with clinical manifestations, imaging examination and so on.

Currently, the gold standard for diagnosing OVCA is a tissue biopsy, which is time-consuming, costly, and carries the danger of tumor spread; thus, a new molecular tool must be developed to enhance the detection rate of OVCA and the survival rate. As an early non-invasive diagnostic and therapeutic approach, liquid biopsy can detect tumor cells or tumor cell DNA fragments with high activity and immune evasion in peripheral body fluids such as blood, urine, and cerebrospinal fluid (35). In fact, circulating tumor cells (CTCs) is a kind of tumor cell that escapes from the primary tumor or metastasis to the peripheral blood circulation and survives, which is closely related to tumor metastasis and proliferation; ctDNA refers to the genetic material released by tumor cells after death or rupture, which mainly exists in the circulatory system of tumor patients too. It can also be used to monitor tumors in a real-time and dynamic manner. As expected, CTCs and circulating tumor DNA (ctDNA) have been extensively studied, and research has revealed that they are closely associated with the tumor burden but are insensitive to the early stage of cancer (36). However, viable tumor cells, apoptotic cells, and cell fragments can be found in peripheral blood, causing inaccuracies in CTC detection results.

Furthermore, ctDNA is fragmented, extremely low in concentration, and has a short half-life, necessitating great specificity and sensitivity in the detection technology (37). In recent years, there have been an increasing number of studies on exosomes, and the proteins carried on the surface of exosomes have the fingerprint characteristics of their mother cells, as the characteristics of the abnormal exosome subsets can be identified early through the analysis of proteins carried on the surface of exosomes (38). However, the existing technology makes it challenging to assess the fingerprint characteristics of a single exosome.

In the study, we used the PBA technique to evaluate the EVs derived from ascites and then categorized them based on the characteristics of proteins on the exosome membrane. PBA has the following advantages: (1) resolution of single exosome; (2) not limited by exosome size; (3) affinity capture of exosome without purification; (4) protein detection of single molecule sensitivity; (5) histological data of ultra-multi-factor simultaneous detection; (6) high-throughput detection of all single exosomes in samples. It was revealed that the sub-clusters of CDCP1+ EVs increased in the EVs derived from OVCA ascites. The experiment of the verification group confirms the significant expression of CDCP1 in OVCA ascites exosomes. It is hypothesized that it can be utilized as a marker for OVCA screening. The tissue microarray revealed that patients with elevated CDCP1 expression had a bad prognosis. Previous research has demonstrated that CDCP1 plays a vital role in tumor metastasis and invasion (39). However, this study has no statistically significant difference between distant metastases. We suspect that the outcomes of this study may be attributable to the insufficient sample size. In addition, we have collected ascites and serum samples from women with OVCA and women without cancer, but the expression of CDCP1 is substantially different. Exosomes from OVCA cells are most abundant in ascites, whereas exosomes from other cell origins are somewhat depleted in serum, which may explain the analysis results. Currently, the precise mechanism of CDCP1 in the metastasis and invasion of OVCA is unclear, which is an issue worthy of investigation.

Shortly, our study identifies a novel screening marker and therapy target for early identification and treatment of OVCA, which will significantly increase the positive rate of an OVCA diagnosis. Exosomes can also be employed to treat metastatic or recurrent OVCA; however, this study is still in its infancy and requires additional clinical investigation.

## Data availability statement

The data presented in the study are deposited in the Figshare repository, accession address DOI: 10.6084/m9.figshare.23544642, found at - https://figshare.com/articles/dataset/_strong_Ascites-derived_CDCP1_extracellular_vesicles_subcluster_as_a_novel_biomarker_and_therapeutic_target_for_ovarian_cancer_strong_/23544642.

## Ethics statement

The studies involving human participants were reviewed and approved by Medical Ethics Expert Committee of Zibo Central Hospital. Written informed consent for participation was not required for this study in accordance with the national legislation and the institutional requirements. The animal study was reviewed and approved by Medical Ethics Expert Committee of Zibo Central Hospital.

## Author contributions

JL, LK, and YC contributed to conception and design of the study. FX and SZ organized the database. ZG and DW performed the statistical analysis. LK wrote the first draft of the manuscript. YY, PT and TL wrote sections of the manuscript. All authors contributed to the article and approved the submitted version.

## Supplementary material

Supplementary Table 1 The expression of 113 proteins in each sample.
